# A qualitative study of health professions curricula and management of lateral ankle ligament sprain demonstrates inconsistency

**DOI:** 10.1186/s12909-020-02013-8

**Published:** 2020-03-31

**Authors:** Toni Green, Grant Willson, Kieran Fallon

**Affiliations:** 1 ANU College of Health and Medicine, Australian National University, Acton, ACT Australia; 2 Discipline of Physiotherapy, University of Canberra, Bruce, Australian Capital Territory 2617 Australia

**Keywords:** Curricula, Lateral ankle ligament sprains, Clinical practice guidelines, Ottawa ankle rules

## Abstract

**Background:**

Health educators aim to graduate students who are safe, effective and practice evidence-based medicine (EBM). Clinical Practice Guidelines (CPGs) are tools for translating evidence into clinical practice for health professionals and educators who lack time to appraise the evidence. There have been CPGs published for lateral ankle ligament sprains (LALS) for physiotherapists, nurses, and doctors. Clinical decision rules have also been developed for LALS to increase the safety of practice. The Ottawa Ankle Rules (OAR) were developed to screen for the need for an x-ray following an ankle or foot injury.

**Methods:**

Educators from the Australasian College of Sports and Exercise Physicians (ACSEP), St John Ambulance first aiders, pharmacy, nursing, and physiotherapy disciplines were participants in this study. Using purposeful sampling with semi-structured questions and a LALS case study, 19 Australian educators were interviewed. Curricula and textbooks were also collected and analysed. Two researchers independently analysed the data using a deductive method.

**Results:**

Analysis found that no educator used a CPG to inform their teaching. There was no common LALS curriculum for the five groups studied. There were two approaches: a triage curriculum (St John Ambulance, pharmacy, nursing) and a reflective curriculum (ASCEP and physiotherapy). Textbooks influenced curriculum for physiotherapy, pharmacy and first aid educators. The triage curricula recommend rest, ice, compression and elevation (RICE) alone, while the reflective curricula uses OAR, RICE, immobilisation if the LALS is severe, functional support (brace), exercises and manual therapy. In addition, ACSEP and physiotherapy do not recommend electrotherapy. All five groups were cautious about the use of non-steroidal anti-inflammatory drugs (NSAIDs).

**Conclusions:**

Physiotherapy and ACSEP educators teach OAR. Despite not using the CPGs to inform curriculum, physiotherapy and ACSEP have unintentionally aligned their curriculum with current LALS CPG recommendations. However, nursing, pharmacy and first aid trainers are not teaching OAR or aligned with LALS CPGs. Educators in pharmacy, nursing and first aid should re-examine their curricula and consider possibly teaching OAR and using CPG. Clinical practice guideline developers should consider pharmacists and first aiders as users of their LALS CPGs.

## Background

Health curriculum requires a balance of evidence based medicine (EBM) [1] clinical skills, a positive student experience and consideration of contemporary social/cultural context [2–4]. Choosing content involves educators assessing the quality of resources such as randomised clinical trials, systematic reviews, textbooks, diagnostic studies, and clinical practice guidelines (CPGs), investigating current professional practice and utilising feedback from students, recent graduates and employers [5–7].

Acute lateral ankle ligament sprains (LALS) are a common injury [8, 9] resulting in a significant impact on health [10]. LALS are managed by different disciplines including doctors, physiotherapists, pharmacists, nurses and first aid officers. LALS have been researched extensively with many studies investigating the best evidence for management [11–21], including several Cochrane Reviews [22–25]. There are a number of published LALS CPGs for doctors, nurses, and physiotherapists [26]. A systematic review appraised the quality of seven LALS guidelines and found the overall quality of these CPGs are poor and out of date [26]. A recent LALS CPG [27] published after the inclusion of studies in the systematic review, updates the 2012 Dutch guideline [28]. This guideline is written for doctors, nurses, physiotherapists, and sports masseurs. The guideline development process of this CPG included extensive database searches for high quality studies published in Dutch, English, German, French, Spanish, Danish or Swedish. It is surprising that no published CPGs exist for community pharmacies (pharmacists, pharmacy assistants and shop assistants) or first aid officers. It is common for acute LALS patients to seek advice at pharmacies. In a New Zealand study 96 % of pharmacists recommended RICE (rest, ice, compression, elevation) and saw a mean of nine acute LALS per month [29].

Clinical decision rules have also been developed for LALS to increase safety of practice. The Ottawa Ankle Rules (OAR) screen for the need for an x-ray following an ankle or foot injury [30]. OAR are evidence based practice decision tool [31] and are included in LALS CPGs [27]. Despite the extensive research and development of OAR and LALS CPGs, complications are common following a LALS [32–36]. The high prevalence of negative outcomes post ankle injury question whether OAR and CPGS are included in health professional’s curricula or are clinicians not adhering to the OAR and /or the CPGs. The research question for this study was:

Do educators of Australasian College of Sports and Exercise Physicians, St John Ambulance first aiders, pharmacists, nurses, and physiotherapists use OAR and LALS CPGs in their own curriculum?

## Methods

### Theoretical framework

Understanding the content of LALS curriculum is well suited to a qualitative method of enquiry. This study used deductive thematic analysis that allows themes to be found and interpreted [37]. The study was reported according to the Consolidated Criteria for Reporting Qualitative (COREQ) studies checklist [38]. The study had ethical approval by Australian National University Human Ethics Committee (Protocol number 2016/085).

### Participants

The inclusion criteria for participation was an educator of LALS management. Educators were recruited from the Australasian College of Sports and Exercise Physicians (ACSEP), St John Ambulance and Australian universities via email, sourced from an Internet search and by contacting the organisations for volunteers. Purposive sampling was used to select participants from diverse backgrounds, this was achieved by having educators from a number states and territories of Australia and a number of universities. Once consistent themes appeared for each educator group, one further interview was conducted to confirm themes.

### Data collection

Following screening to confirm eligibility and receipt of written informed consent, semi-structured interviews were conducted face to face or by telephone, according to the participant preference. Participants were asked to make available to the interviewer written curricula and textbooks, explanation was sought on how they use these resources in their teaching of LALS. The first author conducted all interviews. These interviews had a standard set of questions and case study (see Supplement 1). Data collection and analysis were completed concurrently so findings from early interviews informed later interviews, enabling in-depth exploration of evolving themes. Interviews were digitally audio recorded, transcribed verbatim, and were supported by field notes.

### Data analysis

The interviewer (TG) is a physiotherapist with clinical, teaching and research experience. The other researcher (KF) is an Australasian College of Sports and Exercise Physician and was on the St John Ambulance advisory panel. The other researcher (GW) is a physiotherapist with no connection to any participant. One researcher (TG) entered the transcripts and curriculum documentation into NVivo^1^. The interviews, curriculum documentation and textbooks were read and reread many times. Each idea or concept appearing from the data was coded into a node and descriptive memos written to record the researcher’s thoughts and interpretations in NVivo^1^. The coding phase was repetitive, with many levels of analysis occurring as data were coded and constantly compared. Each of the nodes were compared to the eight semi- structured questions and tabulated by professional group in a node abstraction form in Microsoft Excel (2016). This enabled the researcher to conceptualise patterns in the answers to the questions, allowing for themes to appear. To increase internal consistency, the coder recoded the same data twice with a period of 3 months between coding. Another author (KF) read ten interviews and checked that the coding and themes were consistent. Three researchers (TG, KF and GW) then met to compare themes and discussed similarities and differences and agreed on the key themes. Data triangulation through diverse sources from multiple professions and mix of interviews, curriculum notes and textbooks enabled a comparison of different viewpoints.

Participant demographic data were described using means and percentages. It is recognised that qualitative research is difficult to avoid personal bias, therefore, information about the research team has been supplied for credibility.

## Results

### Participants

Nineteen educators consented to be interviewed. The interviews were 30 to 60 min in duration. The characteristics of the participants are shown in Table 1**.** All physicians (3) taught registrars and medical students in Victoria or the Australian Capital Territory (ACT) and were also clinicians.

**Table 1:** Characteristics of the participants

Professional Group (number of participants in that role)	Gender (male %)	Qualification includes PhD (%)	Average age of group (years)	Also Practicing Clinician (%)
Fellows of ACSEP (3)	100	0	59	100
St Johns Ambulance First Aid Trainer (3)	100	0	55	100
Academic Pharmacist (4)	25	75	41	25
Academic Nurse (1), Academic Emergency Nurse (1), Nurse Practitioner (1)	33.3	66.7	55	33.3
Academic Physiotherapist (5), Physiotherapy Clinical Educator (1)	66.7	66.7	42	50

The trainers from St John Ambulance (3) each teach around 7000 students per year and worked as first aiders. These trainers had worked in every state and territory in Australia except South Australia and Tasmania. All three had, in addition to being St John Ambulance educators, trained in Paramedicine.

All of the pharmacists (4) had worked in three or more states and territories in Australia and all states and territories were represented. They had trained in a different university. They had been trained in New Zealand, New South Wales, Tasmania, and South Australia. The pharmacists had taught at a university in the ACT, Northern Territory (NT) and Tasmania. One pharmacist in addition to teaching also worked as a clinician on a part time basis in the ACT.

All nurses (3) taught at universities and had many years of experience as clinical nurses and educators. All nurses had worked in Australia and another country specifically the United States of America and the United Kingdom. The nurse practitioner also taught nurse practitioners in addition to general nurses in hospitals. The emergency nurse was trained in America and had extra credentials in emergency medicine in addition to those of nursing.

The physiotherapists (6) had taught in six different universities. Three male physiotherapists also worked part time as a practicing clinician. As a group the physiotherapists had worked in 3 or more states and territories in Australia. The physiotherapists had been trained in New South Wales, Western Australia, Victoria, and Queensland. One academic physiotherapist had international training as a physiotherapist in Canada and another in the United Kingdom. These two physiotherapists had also worked in these two countries.

### Do educators of Australasian College of Sports and Exercise Physicians, St John ambulance first aiders, pharmacy, nursing, and physiotherapy use OAR and LALS CPGs in their curriculum?

Physiotherapy and ACSEP educators teach OAR and do not use LALS CPGs to inform their curriculum. The educators in nursing, pharmacy and first aid trainers are not using OAR or LALS CPGs. The educators reported two distinct curricula themes: triage and reflective. Nursing, St John Ambulance first aiders and pharmacy supply a one-off triage service. These three professions would encourage the patient to seek further assessment and management. The physicians and physiotherapists reflective curricula incorporate the use of OAR and grading of severity. The physicians source their curriculum from researching databases and reading systematic reviews. The physiotherapists in this study sourced their curriculum from the textbook [39] provided. No educator refereed to a LALS CPG to inform their curriculum.

### Triage curricula

On interview, all St John Ambulance educators agreed there was a set curriculum and that they did follow it. St John Ambulance officers always work in pairs for safety and to consistently give the same LALS management. The curriculum resources analysis confirmed the interview responses. An acute LALS would be used as an example of the generic management of sprain and strain. The patient must be stabilised, immobilised and advice given on rest, ice, compression, elevation, and referral (RICER). If the patient was willing, ice would be placed on the ankle while education was given. They also recommended not to take non-steroidal anti-inflammatories (NSAIDs) as it may delay healing. On questioning about OAR, St John Ambulance educators did not know, use, or teach OAR.

The academic pharmacists all agreed there was a set curriculum LALS assessment activity and supplied a brief description of what they teach. They all recommended RICE and advice on the proper use of medicines. The pharmacists supplied the resources that inform their teaching. In these resources an acute LALS would be used as an example of the generic management of sprain and strain.

*“No, definitely not systematic reviews. I think it would have been like a textbook, and first aid book that was issued by St Johns rest, ice, compression, elevation.” Pharmacy Participant 19.*

All sources and interviews consistently showed that pharmacists do not teach or use the OAR. The pharmacy curriculum was consistent with the textbook [40] provided. Three pharmacy educators from three different universities described a specific tutorial case study of LALS that the students will role play and is used as an assessment of oral counselling skills for sprains and strains.

“*we don’t usually encourage them to touch, but just to see is the bone sticking out, how red it is, how swollen it is. And offer assistance, customer service chair, sit down. Once they’ve done the differential diagnosis to be 100% sure it’s just a sprain then they go through the treatment.” Pharmacy Participant 2.*

All nurses agreed there was no set curriculum. An acute LALS would be used as an example of generic sprain management.

*“general, with lectures and in theory, it’s just a broad soft tissue.” Nurse Participant 11.*

They teach the students to encourage weight bearing and RICE and if the pain was too severe within 24 h to go to a doctor. Two out of three nurses knew the OAR but attributed that to their further training and it is not taught to nurses. However, nurse practitioners are diagnosing LALS using diagnostic ultrasound.

*“The nurse is there specifically to look at the patient, understand what’s going, and especially a triage nurse, to make sure that there’s nothing significant happening. But they’re not there to do a drawer test (physical examination), they’re not there to specifically diagnose, That’s the nurse practitioner role, or the medical role.” Nurse Participant 18.*

*“Yeah, there’s lots of states that nurse practitioners use ultrasound” Nurse Participant 18.*

### Reflective curricula

The physicians all agreed there was no set LALS curriculum and teaching/learning activities and that they did not use LALS CPGs as a teaching resource. They do teach the Ottawa Ankle Rules (OAR).

*“I don’t have a set curriculum* per se*, I just use, you know, experience I guess.” Physician Participant 1.*

All physicians taught differential diagnosis of bone or syndesmosis injuries to separate these from a sprain. They consistently described teaching management that included RICE, referral to a physiotherapist, and the importance of management of a severe sprain with a period of immobilisation in a boot. Medication was discussed and all physicians taught their students to weigh up the risks and benefits of NSAIDs.

*For pain relief maybe. There’s a risk of, you know theoretically a risk of increasing bleeding. Physician Participant 1.*

When instructing their students, the physicians were specific and consistent in their process of reflective practice. One physician instructed his medical students and registrars to use ultrasound to confirm their LALS diagnosis. In addition, he was teaching stress tests with the aid of the ultrasound scanner.

*“So, I sit down there and look at how they assess the problem, and I look at how they assess the problem from the history, and then their examination, and then what they… what decisions they make after that.” Physician Participant 9.*

**Fig. 1:**
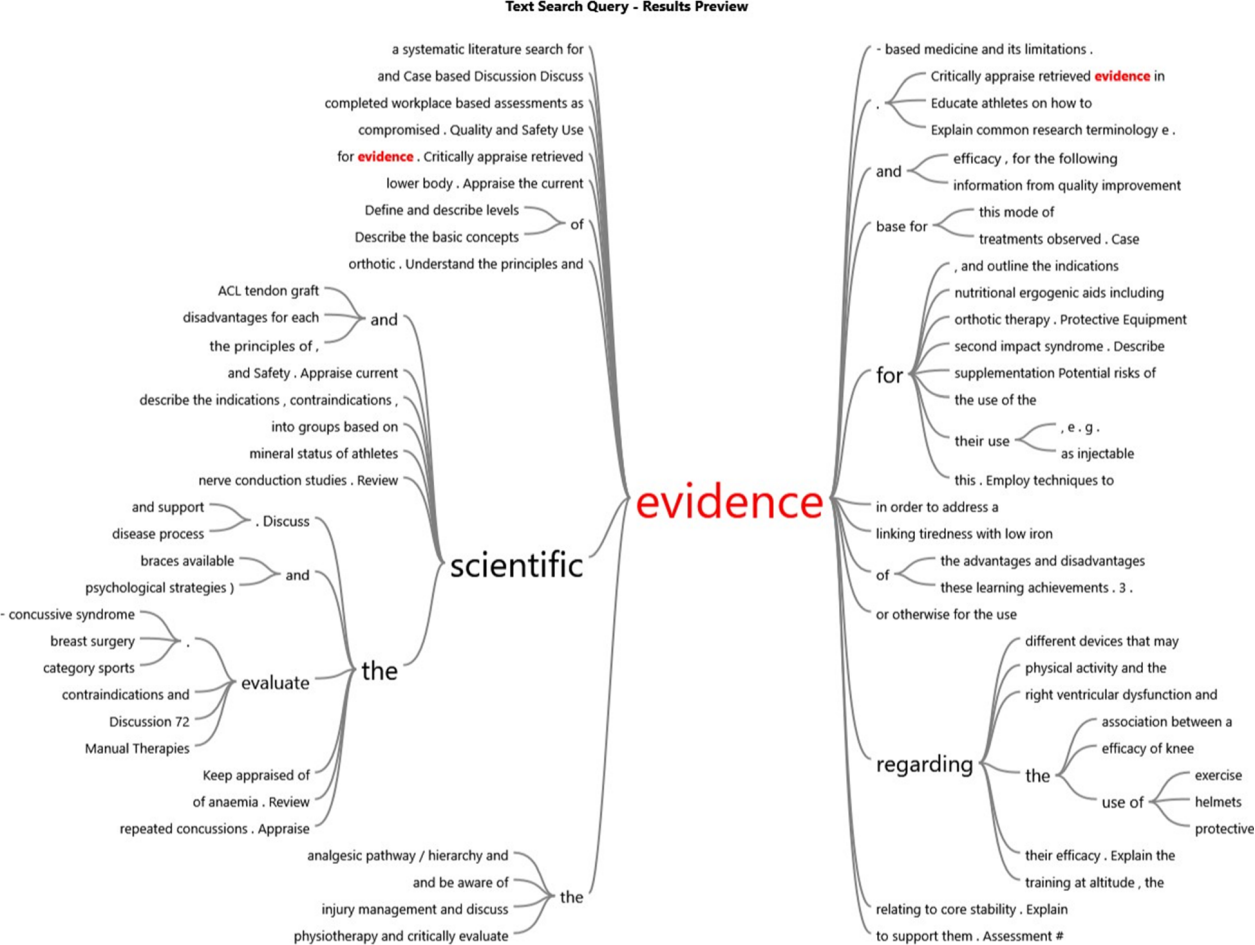
Word Map for term “evidence” text search query for ACSEP 2017 Curriculum and Tutorial Program using NVivo (NVivo 11. Version 11 2015. QSR International Pty Ltd)

This theme of independent ability to research is repeated 35 times in the ASCEP 2017 Curriculum and Tutorial Program Version 6 and includes a list of tutorial topics but not specific content or specific LALS management advice. Instead it consistently shows that evidence should be appraised and discussed at the tutorial. The physicians must have the skill set to be able to search the clinical literature to answer a clinical question. This linking of evidence with scientific and critical appraisal can be seen in the Word Map (Fig. 1). The figure was created using NVivo^1^ query tool with exact matches on the word “evidence” for ACSEP 2017 Curriculum and Tutorial Program. The figure visually shows that the more frequent the word is used the font becomes larger. In addition, it shows the phrases associated with the word “evidence.” The word map shows the physicians process of designing their curriculum and teaching activities.

The physiotherapists all agreed there was no set curriculum and that they did not use CPGs as a teaching resource. One participant described he would search the Cochrane Library of Systematic Reviews to support his teaching of certain recommendations for LALS management [25].

“*I’m pretty sure that there’s systematic review that talks about complete immobilisation versus functional braces ….. long term I don’t think there was a difference, but short term it was better to have the functional brace.” Physiotherapist Participant 4.*

All described teaching students to take a history, physical examination, special tests including OAR, differential diagnosis of bone and syndesmosis injuries. They consistently taught detailed management that included RICE, and the importance of management for a severe sprain to include a period of immobilisation in a boot. One academic acknowledged that they were teaching a new acronym: protect, optimal loading, ice, compression, and elevation (POLICE) rather the RICE.

On questioning about medication, physiotherapy educators were aware of the conflict to prescribe or not NSAIDs.

*I mean we would advise her to discuss with a pharmacist or a doctor ‘cause we’re not officially prescribing profession. Physiotherapy Participant 5.*

## Discussion

The themes from this study show the complexity of implementing a common LALS curriculum. The triage curriculum does not instruct students to use OAR or CPG recommended interventions. The physicians include greater detail in teaching the process of EBM. In this study all physicians were clinicians as well as educators and were on average the oldest age group. The inherent nature of speciality in medicine and ASCEP process means that these educators spend more time with their patient over an extended period and this leads to assessing what interventions work well, the practice of reflection and by completing the five steps of EBM [1]. Clinicians learn from complicated cases and failures, converting the need for information to generate an answerable research question, they then search databases, critically appraising the literature and then integrating it into clinical medicine (EBM).

When clinicians learn from their patients it is counted as evidence in EBM [41]. In comparison, the triage curriculum does not allow collection of evidence from the patient. Education to professions who use a triage curriculum of the severity of LALS and the complications that may occur if strengthening and balancing exercises are not done, needs to be addressed.

The pharmacy educator’s curriculum was influenced by textbooks and First Aid manuals. This is supported by a recent systematic review that found that pharmacists were frequently consulted for advice on managing sprains, but their advice was not always being guided by current best evidence [42]. It is possible that updates in textbooks to include recent recommendations [26, 27] may influence curriculum in the future.

No physiotherapy educator was teaching medication management for their LALS patients due to legislation in Australia. The 2018 LALS CPG recommends that non-steroidal anti-inflammatory drugs (NSAIDs) may be used by patients who have incurred an acute LALS for the primary purpose of reducing pain and swelling [27]. It is possible in the future that Australian physiotherapy curriculum may include pharmacology teaching related to these medications as in other physiotherapy curriculum for example in the United Kingdom [43].

An unexpected outcome of this study was that some physicians and nurse practitioners are using ultrasound to diagnose LALS. The American Society for Sports Medicine has recently recommended a sports ultrasound curriculum for sports medicine fellowships [44]. Searching the literature shows that physiotherapists are using ultrasound to diagnose the amount of swelling for LALS [45]. Ultrasound has also been shown to be inexpensive and quick to visualise soft tissues and fractures [46]. Future research could assess the feasibility, validity, and reliability of the use of ultrasound within the scope of practice of physiotherapy.

## Conclusions

Physiotherapy and ACSEP educators teach OAR. Despite not using the CPGs to inform curriculum, physiotherapy and ACSEP are aligning their curriculum with LALS CPG. However, nursing, pharmacy and first aid trainers are not using OAR or LALS CPG. Educators in pharmacy, nursing and first aid should re-examine their curricula and possibly teach OAR. More research is needed to assess if teaching OAR to pharmacy, nurses and first aiders is feasible. Clinical practice guideline developers may consider pharmacists and first aiders as users of their future LALS CPGs.

## Supplementary information

**Additional file 1.** Supplementary 1: Interview questions and case study.

## Data Availability

The datasets generated and/or analysed during the current study are not publicly available due to restrictions of Australian National University’s Human Research Ethics Board that does not allow sharing of data collected as part of research approved by the Board.

## Abbreviations

ACSEP: Australasian College of Sports and Exercise Physicians
COREQ: Consolidated Criteria for Reporting Qualitative
CPD: Continuing professional development
CPGs: Clinical Practice Guidelines
EBM: Evidence-based medicine
LALS: Lateral ankle ligament sprains
NSAIDs: Non-steroidal anti-inflammatory drugs
OAR: Ottawa Ankle Rules

## Footnotes

1 NVivo 11. Version 11 2,015. QSR International Pty Ltd
