# Supplementary material for: A qualitative study of health professions curricula and management of lateral ankle ligament sprain demonstrates inconsistency
Source: BMC Med Educ. 2020 Mar 31;20:99. doi: 10.1186/s12909-020-02013-8 (PMC7110746; doi:10.1186/s12909-020-02013-8)
Supplement: Supplementary file 1 — Additional file 1. Supplementary 1: Interview questions and case study. [file 12909_2020_2013_MOESM1_ESM.docx]

1. Do you teach students how to manage acutely sprained ankles?

2. If yes, please describe the specific management that you teach at your institution or organisation?

3. If no, what is the current curriculum for acute sprained ankle management at your institution or organisation?

4. Could the research team have a link to or a hard copy of the curriculum?

5. The chief investigator will provide a case study of a de-identified sprained ankle client's subjective and objective findings and then ask how this profession would manage this client?

6. Do you think that clinicians in your field manage acutely sprained ankles as taught by their training facilities?

7. If no, what are the barriers you perceive for evidence based management?

8. If yes, what are the enablers you perceive for evidence based management?

1/08/16 Patient: Susie Smith: DOB: 17/1/2000

**ASSESSMENT: √**


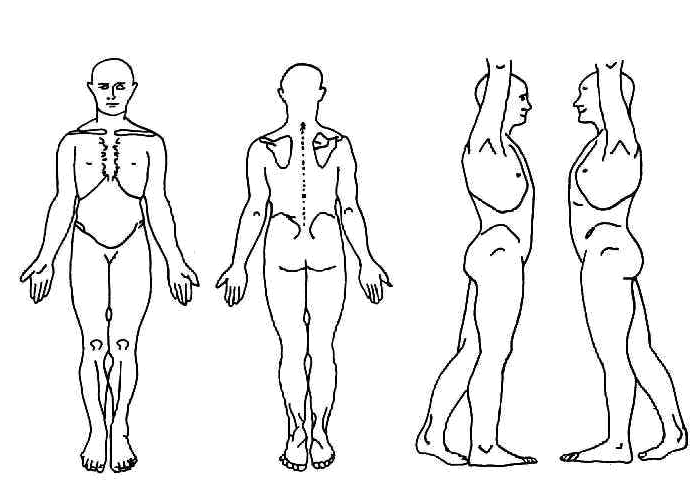


**√**

**√**

**√**

**√**

Pa: sharp, deep, intermittent 0-6/10

Susy 16 years old netballer, rolled her ankle and sprained her right ankle yesterday while playing netball. This is the first time she has hurt her ankle. She has never had an injury before.

Wants to play on Saturday and in 8 weeks (long term goal) is a round robin weekend of netball, desperately wants to play for her club. X-ray NAD. Subjective all clear no red (Ottawa Rules are OK ) or yellow flags. Special questions all good. On crutches NWB, holding foot in plantarflexion (AROM = PROM 20 degrees, dorsiflexion (AROM = PROM 2 degrees), scared to move foot, very sore to touch.

Tender over ATFL.

**INTERPRETATION:**

Provisional diagnosis:

**PLAN/Problem List/goals:**

**Short Term:**

1.

2.

3.

**Long Term:**

1.

2.

3.

**INTERVENTION TODAY:**

1.

2.

3.

**EVALUATION:**

1.

2.

3.
